# Supplementary material for: Development of Smallpox Antibody Testing and Surveillance Following Smallpox Vaccination in the Republic of Korea
Source: Vaccines (Basel). 2024 Sep 26;12(10):1105. doi: 10.3390/vaccines12101105 (PMC11510797; doi:10.3390/vaccines12101105)
Supplement: Supplementary file 1 [file vaccines-12-01105-s001.zip › vaccines-3178626-supplementary.pdf]

Table S1. Result of ROC Analysis of PRNT

Cut-off (Endpoint titer)	Sensitivity (%)	1-Specificity (%)
> 64	0	100
> 32	10	100
> 16	35	100
> 8	60	100
> 4	100	100
> 2	100	100
> 1	100	0

Table S2. $PRNT_{50}$ Values before and after vaccination for 20 individuals serum

Subject(serum)	Pre serum*	Post serum**	Fold increase
A	< 2	32	32
B	< 2	8	8
C	< 2	64	64
D	< 2	8	8
E	< 2	16	16
F	< 2	64	64
G	< 2	8	8
I	< 2	32	32
J	< 2	32	32
K	< 2	8	8
L	< 2	8	8
M	< 2	8	8
N	< 2	16	16
O	< 2	32	32
P	< 2	16	16
Q	< 2	16	16
R	< 2	8	8
S	< 2	8	8
T	< 2	16	16
U	< 2	32	32

* Before vaccination

** 4 weeks after smallpox vaccination

※ Titer of Normal human serum (Sigma) was less than 2.

Table S3. Result of ROC analysis for IgG ELISA using A27L recombinant antigen

Normalization method	Dilution factor	Cut-off	Sensitivity (%)	Specificity (%)	AUC
	1:50	> 0.7575	100	95	0.993
	1:100	> 0.4455	100	95	0.993
ΔO.D._1	1:200	> 0.1427	100	75	0.948
	1:400	> 0.1066	100	75	0.923
	1:800	> 0.0706	100	75	0.905
	**1:50**	**> 0.4667**	**100**	**100**	**1.000**
	1:100	> 0.4768	100	95	0.990
ΔO.D._2	1:200	> 0.3769	100	85	0.965
	1:400	> 0.1413	100	80	0.930
	1:800	> 0.0943	100	75	0.908

Table S4. Result of ROC analysis for IgG ELISA using whole vaccinia virus

Normalization method	Dilution factor	Cut-off	Sensitivity (%)	Specificity (%)	AUC
ΔO.D._1	1:50	> 0.2142	100	70	0.878
	1:100	> 0.0371	100	90	0.960
	1:200	> 0.0897	100	90	0.975
	1:400	> 0.0230	100	85	0.970
	1:800	> 0.0270	100	85	0.975
ΔO.D._2	1:50	> 0.0752	100	90	0.950
	1:100	> 0.0202	100	90	0.958
	1:200	> 0.0996	100	95	0.978
	**1:400**	**> 0.0535**	**100**	**95**	**0.983**
	1:800	> 0.0377	100	85	0.977

Standardization of ELISA results

Two methods were employed to standardize the variation in color development across plates. One method [18] (Method 1) involved establishing a blank well in each plate, computing the average value, and subsequently adding three times the standard deviation to derive the ΔO.D._1 value. The alternative method [19] (Method 2) involved subtracting the average O.D. value of normal human serum from the O.D. value of the sample to obtain ΔO.D._2. These approaches were used to normalize color development variations across plates.

Receiver operating characteristic curve analysis

The diagnostic accuracy of standardized ΔO.D. values was assessed using receiver Operating Characteristic (ROC) curve analysis using MedCalc version 22 software

(Belgium). ROC curve analysis was used to determine the optimal cut-off value based on the relationship between sensitivity and specificity. The area under the ROC curve (AUC) represents the overall accuracy of the diagnostic test, with larger AUC values indicating heightened accuracy. Ranging from 0 to 1, the AUC value closer to 1 signifies perfect diagnostic capability, whereas a value of 0.5 denotes performance equivalent to random guessing. This methodology facilitated the establishment of a cut-off value for the specific diagnostic test, evaluating its utility through the computed sensitivity and specificity at this threshold.
